# Comprehensive Evaluation on Space Information Network Demonstration Platform Based on Tracking and Data Relay Satellite System

**DOI:** 10.3390/s20185437

**Published:** 2020-09-22

**Authors:** Feng Liu, Dingyuan Shi, Yunlu Xiao, Tao Zhang, Jie Sun

**Affiliations:** Department of Electronic and Information Engineering, Beihang University, Beijing 100191, China; liuf@buaa.edu.cn (F.L.); dingyuanshi@buaa.edu.cn (D.S.); yunlu.xiao@buaa.edu.cn (Y.X.); sunjiehyit@buaa.edu.cn (J.S.)

**Keywords:** space information network, tracking and data relay satellite, comprehensive evaluation index system, analytic hierarchy process

## Abstract

Due to the global coverage and real-time access advantages of the Tracking and Data Relay Satellite System (TDRSS), the demonstration platform based on TDRSS can satisfy the new technology verification and demonstration needs of the space information network (evolution from sensorweb). However, the comprehensive evaluation research of this demonstration platform faces many problems: complicated and diverse technical indicators in various areas, coupling redundancy between indicators, difficulty in establishing the number of indicator system layers, and evaluation errors causing by subjective scoring. Concerning the difficulties, this paper gives a method to construct this special index system, and improves the consistency of evaluation results with Analytic Hierarchy Process in Group Decision-Making (AHP-GDM). A comprehensive evaluation index system including five criterions, 11 elements, more than 30 indicators is constructed according to the three-step strategy of initial set classification, hierarchical optimization, and de-redundancy. For the inconsistent scoring of AHP-GDM, a high-speed convergence consistency improvement strategy is proposed in this paper. Moreover, a method for generating a comprehensive judgment matrix (the aggregation of each judgment matrix) aggregation coefficient is provided. Numerical experiments show that this strategy effectively improves the consistency of the comprehensive judgment matrix. Finally, taking the evaluation of TDRSS development as an example, the versatility and feasibility of the new evaluation strategy are demonstrated.

## 1. Introduction

The information obtained by satellites’s sensors account for an increasing proportion of the total information obtained by humans. Then, the sensorweb proposed by NASA, has created an interoperable environment for a diverse set of satellite sensors via the use of software and the Internet. The authors of [1] point out that the development trend of sensorweb is integrating with other networks. The space information network is the high-level result of the further integration of sensorweb and space-based networks [2]. As a result of the sensorweb‘s evolution, the space information network is a network system that acquires, transmits, and processes space information in real-time based on a space platform (such as a synchronous satellite, a medium or low orbit spacecraft, a near space vehicle, a drone, a manned aircraft, etc.) [3]. With the construction and development of the space information network (SIN), several researches about space network models, network information theory, space information cognition, and space network security have been proposed [4,5]. However, a comprehensive demonstration and verification systems for these new results are still urgently required. In recent years, a consensus has been formed on the Tracking and Data Relay Satellite System (TDRSS) as the backbone network of SIN [6]. As an integrated system in orbit, TDRSS is similar to the space information network to a certain extend regarding its structure, function, and service objects, which can provide a feasible method to the demonstration of space information network [7]. The comprehensive integrated demonstration platform built with TDRSS as the core can be used as a basic network architecture for the current demonstration of space network integration demonstration. It has the advantages of low implementation cost, strong demonstration ability, high scalability and flexibility.

During the construction of a demonstration platform based on TDRSS, on the one hand, it is necessary to consider the evaluation of the demonstration platform itself. On the other hand, new technology test results need new evaluation models and method support. One or two indicators cannot meet the above needs. Therefore, comprehensive evaluation with multiple indicators for the demonstration platform needs to be studied and designed.

The comprehensive evaluation study will face many problems. First, due to the wide range of technical fields involved in the demonstration platform itself and the diversity of applications of the demonstration, the platform-related indicators are numerous and diverse. Second, the attributes of some indicators might overlap, have strong correlation or even repetition, leading to coupling redundancy. Then, as a typical tree structure, the indicator system may have too many lower-level indicators, causing difficulties in adjusting the number of layers. Finally, the subjective scoring by people caused evaluation errors, which often made the evaluation results unacceptable. Especially when there are too large differences among the subjective scores, how to aggregate the opinions of the experts is also a problem. In the field of decision-making evaluation, the first three problems usually appear in the process of constructing the indicator system, and the last problem is common in the application process of the indicator system.

The solution of the problem above in this paper is as follows. For the first three problems, we construct the index system layer by layer with the logical sequence. First, we use cluster analysis to achieve the classification and selection of indicators to determine the first level indicators (criteria layer). Then, we reconstruct the indicator system’s levels to determine the second level indicators (element layer). Finally, the third level indicators are decomposed and optimized to remove coupled redundant indicators, which completes the index system. Aiming at the last problem, the source of the error is analyzed and evaluated, and a combination of the eigenvector method and the geometric average method is adopted to reduce the generation and transmission of the error.

The specific contributions of this paper are as follows.

We built a comprehensive index system consisting of five criteria, 11 elements, and more than 30 indicators, which effectively satisfied the TDRSS-based demonstration platform’s evaluation needs. A universal construction method from the initial indicator set to the comprehensive indicator system is provided.

We proposed an analytic hierarchy process consistency enhancement strategy suitable for group decision-making/evaluation scenarios. Numerical experiments show that the initial value of the strategy is better and has a faster convergence speed, which can effectively improve the consistency of the comprehensive judgment matrix. In addition, in order to reduce the transmission of error in the aggregation process, a flexible method for determining the aggregation coefficient of group experts is given.

**Figure 1:**
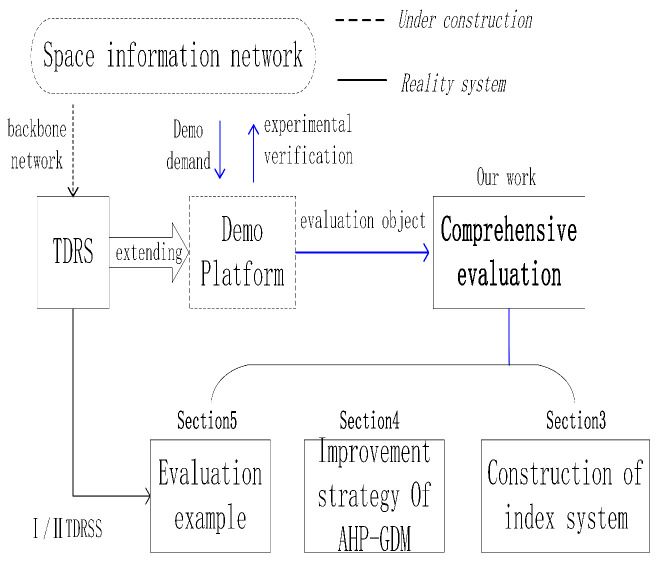
The logical diagram of this paper.

The rest of the paper is organized as follows. Section 2 investigates the research status of the index system construction principles, methods and existing systems, and some new developments of the analytic hierarchy process. Section 3 gives the index system construction’s key procedures of classification, layering, optimization and feedback. The comprehensive evaluation index system for the space information network demonstration platform is proposed based on the above operations. Section 4 studies the subjective error caused by the multi-expert scoring in the classical evaluation method—Analytic Hierarchy Process—in group decision-making (AHP-GDM), and gives an improved consistency enhancement method. Section 5 is a comprehensive evaluation of the effectiveness of the two-generation TDRSS to further verify the practical significance of the evaluation system. A logic diagram of each section is shown in Figure 1.

## 2. Current Works

As the two key issues in comprehensive evaluation, the index system and evaluation method (this article mainly focuses on the AHP) have been extensively studied.

### 2.1. Index System Research

The index system is the most critical part of comprehensive evaluation. Its construction principles are introduced in [8,9]: (1) Completeness—the index system should cover as much attributes of the evaluation object as possible; (2) Measurability—the indicators in the system should be preferably measurable and objective; (3) Independence—the indicators are as unrelated as possible; (4) Simplicity—use as few indicators as possible to cover as many attributes as possible; (5) Hierarchical principle—the system should have a clear hierarchical structure; (6) Accuracy—an evaluation will reveal and convey technically adequate information about the features. Among them, Completeness, Independence, and Measurability are the three most important principles in the construction of the index system.

There are large diversities due to the different evaluation targets and objects for the construction method and process of the indicator system. Representative solutions, such as those in [10], established a set of comprehensive evaluation index system based on multiple characteristics of evaluation objects. The authors of [11] provided a viable way to construct the index system based on the indicator importance sort algorithm, which has low algorithm complexity and can reflect the architectural constraints in the construction of the index system. The authors of [12] used the “R-cluster + coefficient of variation analysis” method to quantitatively screen the initial indicators, which is an efficient method for indicator selection and classification. The authors of [13] presented a simple and metric methodology for evaluating remote sensing satellite effectiveness, which focuses on product quality parameters, satellite availability, and predicted satellite reliability. The authors of [14] focused on the assessment of coverage effectiveness of remote sensing satellite, and proposed and designed a multi-index evaluation method based on index weight using entropy weight method and analytic hierarchy process. However, at present, there is no universal method to effectively reconstruct the index system’s hierarchy.

As far as the content of the proposed index system is concerned, the Internet Engineering Task Force (IETF) has given a series of authoritative documents for network performance metrics (such as delay, jitter, packet loss, connectivity, etc.) [15] that have laid the foundation of the network evaluation index system. Driven by various evaluation needs, corresponding evaluation index systems have been proposed from ground networks to wireless access networks and to space-based communication systems [16,17,18]. The relevant subsystems and network node equipment of the SIN already have corresponding indicators and indicator systems. They can provide a large number of input indicators for the indicator system of the SIN demonstration platform. However, the platform still lacks a complete set of comprehensive evaluation index systems, which requires urgent research.

### 2.2. Evaluation Method Research

As the most representative evaluation method in the field of comprehensive evaluation, the AHP was first proposed by Dr. Saaty in the 1970s. Because the consistency of the expert score matrix (pairwise comparison judgment matrix) is an important basis for measuring the credibility of the evaluation results, it has been the main research focus of AHP. Based on Frobenius–Perron theorem, Harker first proposed a consistency enhancement algorithm for judgment comparison matrix, which replaces the largest error element by the combination of the main feature vector elements [19]. Later, Dr. Saaty analyzed the influence of the small disturbance on the consistency of the judgment matrix from the matrix principle in [20], and calculated the partial differential matrix and error matrix corresponding to the nearly consistent matrix. Furthermore, an improved judgment matrix correction method is given based on Harker’s algorithm. Aiming at the problem of missing elements in the judgment matrix in emergency decision-making scenarios, the authors of [21] extended the geometric mean-induced deviation matrix to calculate the missing elements and improve the consistency ratio of the judgment matrix at the same time. The missing elements given by this method can also be used to correct the error elements. The authors of [22] presented a theoretical framework and a procedure for revising the judgments and improving the inconsistency of an AHP pairwise comparison matrix, when the Row Geometric Mean (RGM) is used as the prioritization procedure and the Geometric Consistency Index (GCI) is the inconsistency measure.

With the application of AHP in group decision-making and evaluation, not only must the issue of consistency deviation of the judgment matrix must be considered at this time, but also the judgment matrix given by each expert must be effectively aggregated. In the AHP group decision-making (AHP-GDM), the aggregation of individual judgments (AIJ) and the aggregation of individual priorities (AIP) are divided according to the order of aggregation. Among them, AIJ first aggregates the judgment matrix to obtain a comprehensive judgment matrix, and then obtains the feature vector of the comprehensive judgment matrix. Moreover, AIP is to directly aggregate the eigenvalues and eigenvectors of each judgment matrix [23]. The authors of [24], based on the aggregation order of AIJ, proposed a judgment matrix aggregation method based on Hadamard product. In addition, based on the geometric mean method, the authors of [25] created a new decision tool called the Consistency Consensus Matrix (CCM) for consensus building in AHP-group decision making. The authors of [26] presented an extension of the CCM, the precise consensus consistency matrix (PCCM), which can provide more informed and participative GDM and offers more accurate estimations for the group’s priorities. The authors of [27] proposed an iterative algorithm for improving the compatibility of the PCCM, gave a detailed explanation of the process followed to solve the optimization problem, for the consideration of different weights for the decision makers in the calculation of the PCCM. To deal with different levels of consistency when there are a number of decision-makers, the authors of [28] presented some of the methods for defining the individual weights of decision makers in group AHP decision making. The authors of [29] outlined a new approach to aid the consensus decision making process, for which two heuristic algorithms are developed. The first algorithm is designed to assist the decision maker in achieving a predefined consistency level, and the second is designed to achieve consensus while controlling the individual consistency level. Several classical numerical examples are compared to validate the effectiveness of the proposed approach. The aggregation coefficient of those methods only considers the authority of experts, and it cannot fully reflect the impact of the current evaluation error on the final aggregation result.

The authors of [19,20] formed a method that relied on eigenvectors for consistency enhancement (also called, feature vector method). This method has a faster descending speed, but it is more sensitive to the initial value. Especially when the element deviation in the judgment matrix is large or missing, the falling speed of this method will be affected. The authors of [21] proposed a classic method of consistency enhancement based on row geometric mean. Its sensitivity to the initial value is low, but its ability to continuously decrease is weaker than the eigenvector method. We believe that the combination of the two methods can achieve complementary advantages. Although there are mature aggregation methods in the aggregation process of judgment matrices [28], there is still a lack of a balanced strategy of aggregation coefficient that takes into account expert authority and the initial input inconsistency.

## 3. Construction of Comprehensive Index System

**Figure 2:**
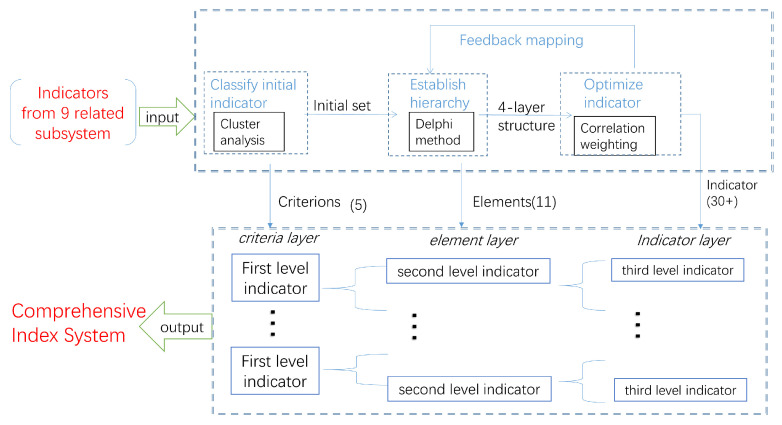
Construction solution of index system.

Instead of a simple overlay of the indicators of the existing subsystems, the comprehensive index system is constructed step by step in a logical sequence in this paper. In this section, the complete process of constructing a unified comprehensive index system from the scattered initial indicator set is shown in Figure 2.

The subsystem indicator sets are used as inputs to gradually implement classification, layering, and optimization. The process is as follows. First, similarities would be found in a large number of initial indicators. Cluster analysis is used to achieve classification and selection of indicators, obtain five types of initial indicator sets, and output first level indicators (criteria layer) of the indicator system. In the second step, on the current three-layer framework, reconstruct the level of the index system and output the second level indicators (element layer) of the index system. The third step is to decompose and optimize the remaining underlying indicators to remove coupling redundancy Other indicators and output third level indicators (indicator layer). After moderately adjusting the mapping relationship between the index layer and the element layer, the construction of the index system is completed.

### 3.1. Investigation, Screening, and Classification of Initial Indicator Sets

In this part, the screening and classification of the initial indicator set are introduced. Five major criteria of the index system are established, which are called 1st level indicators. These five criteria take into account the functional attributes of the SIN and the design requirements of the demonstration platform. First, the SIN-related systems are investigated, and the initial index set is prepared for the index system. Then, analyze the requirements of the SIN demonstration platform to establish classification rules. Finally, cluster analysis is used to classify and verify the correctness of the classification rules.

#### 3.1.1. Investigation of the Initial Indicator Set

We acquire the important input for the index system construction, by investigating the indicators of SIN’s subsystem. Based on previous research, we investigated representative indicators of systems including mobile communication systems, terrestrial IP networks, space-based remote sensing, navigation, and measurement and control, etc. The results are shown in Table 1.

#### 3.1.2. Establishing Indicator Classification Criteria

After the initial indicators are obtained, they need to be classified according to the similarities between the indicators. This commonality is the core criterion of the comprehensive index system and provides classification criteria for subsequent cluster analysis. In this paper, we summarize the commonality from the development needs of the SIN, and then verify its rationality by cluster analysis.

By analyzing the typical application scenarios of relay satellite systems (including space test verification scenarios) and the development trend of SIN, the following requirements are obtained.

- Real-time transmission and rapid response-described by time-domain related indicators.
- High-efficiency and large-capacity-described by frequency-domain and data quality-related indicators.
- Reliability and stability-described by system security-related indicators.
- Airspace and service coverage-described by antenna coverage performance related indicators.
- The measurement requirements required for the demonstration test-described by accurate measurement and simulation-fit related indicators.

With reference to the naming rules of the ADC model of the United States Weapons System Effectiveness Industry Advisory Committee (WSEIAC), the five categories of index criteria mentioned above are named coverage, timeliness, capability, accuracy, and dependability. They comprise the first level in the comprehensive index system.

#### 3.1.3. Commonality Verification with Preliminary Mapping

The core goal of this step is to verify the foregoing classification criteria through cluster analysis. Based on the classification criteria, the indicators and criteria are initially mapped. After mapping, the index system has an initial three-tier structure. The processing steps are as follows.

- Simple screening of hundreds of initial indicators obtained from the survey, then removing indicators that are obviously not related to SIN.
- The selected indicators are then tabulated and distributed to experts for a single round of scoring. The specific score is the distance of a certain indicator compared to the five criteria.
- Using calculation software with system cluster analysis function (such as SPSS) to divide the preliminary selection indicators into several categories. The initial mapping of indicators and criteria is then completed according to the tree diagram after classification.

### 3.2. Establishment and Optimization of Hierarchical Structure

The core work of this section is to follow the empirical principle to establish the appropriate number of structural levels of the index system, so that the “granularity” of the indicators at the same level is uniform. Meanwhile, the outdegree of the indicators with affiliation between the upper and lower levels should be moderate. And in the process, determine the element level indicators, i.e., second level indicators.

After preliminary classification, the current index system has a three-tier structure, which could be described as a three-layer tree. In the practical application, it is usually not desirable for a upper-level indicator to include too many lower-level ones. That means the outdegree of upper level in index system tree should be under-controlled. It is generally considered that an outdegree between 1 and 9 is suitable for the index system. However, aggregating more than forty indicators into five criteria could possibly lead to larger outdegree for certain criteria. In addition, there is also the problem that the range of attributes covered by one indicator, defined as “granularity”, could be largely different among every indicator. It is unreasonable to put indicators with different granularity in the same layer. Moreover, some indicators are considered redundant indicators which have a certain affiliation with other indicators, need to be deleted.

**Figure 3:**
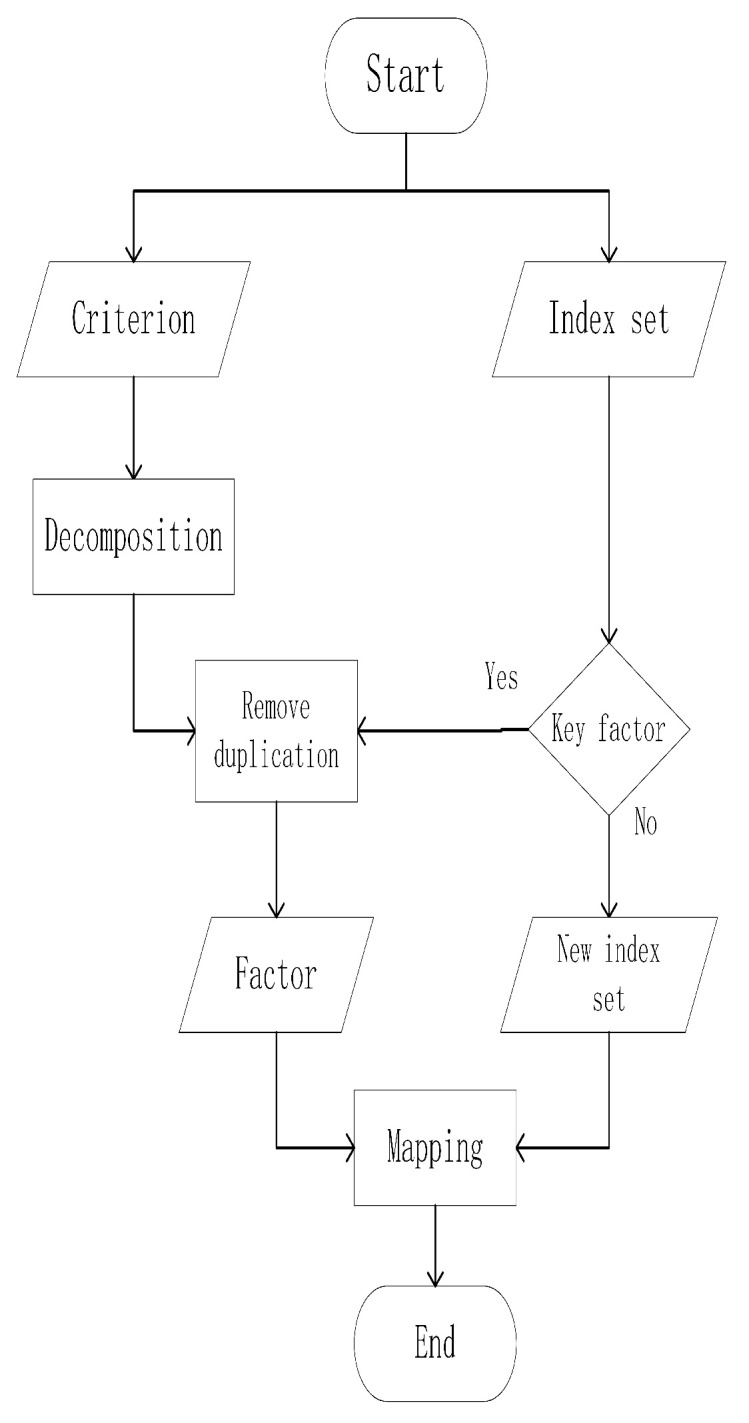
The processing flow of system reconstruction.

In order to solve the above problems of the current three-layer index system, we add an element layer between the criterion layer and the indicator layer, transforming the architecture to four layers. The method of creating the element layer is described as follows; from top to bottom, the evaluation attributes of the criterion, i.e., the physical meaning of the specific representation, are decomposed to obtain the main element layer elements. For example, the timeliness criterion can be further decomposed into two types—the delay of the network and the delay of the platform system. From bottom to top, the criteria of the index layer of the original system are determined as separable. If they are not separable, they will be included in the index layer of the new system. If they are separable, they will continue to be judged. If the system element layer is not an element, the sub-indexes after decomposition are classified into the new index system. The processing flow is shown in Figure 3.

### 3.3. Decomposition and Optimization of Indicators

This section focuses on the problems of redundancy and low recognition in the indicator layer. Each indicator is optimized to meet the basic principles of indicator system construction: independence and testability. Finally, the content of the indicator layer (third level indicators) was determined.

After the criteria layer of the evaluation index system is clearly defined, the following problems still exist in the index layer.

1. The recognition of some indicators is relatively low (identification degree refers to the ability and effect of a statistical evaluation indicator in distinguishing the value characteristics of each evaluation unit in one aspect, so it is also called discrimination degree).
2. There are qualitative indicators that are difficult to characterize numerically.
3. There are redundant and overlapping indicators.

It could be seen that 1 and 2 violate the measurability principle of the indicator system, and 3 is against the principle of independence.

To ensure the measurability of the indicator, it is necessary to enhance the recognition of the indicator or directly remove the indicator with low recognition. A simple and direct method is to use the coefficient of variance of the measured value of the indicator (where the coefficient of variance = Standard deviation/mean) measure the degree of recognition of the index value. For some qualitative indicators that are difficult to quantify, a comprehensive quantitative decomposition method is employed. A single qualitative indicator is decomposed into a plurality of quantifiable and quantitative indicators, such as network handover performance indicators can be jointly characterized by indicators such as signaling overhead, packet loss rate, handover throughput, and handover delay.

In order to ensure the independence and simplification of indicators, redundant and coupled indicators need to be processed. Redundancy in this article refers to the degree of conceptual overlap between indicators. For example, the packet forwarding rate of a router is the same as the actual content of the router’s concept of port throughput. Therefore, one of them can be deleted when used simultaneously. The coupling degree mainly measures the degree of positive/negative correlation between indicators. Redundant indicators are reduced by adjusting the Comprehensive weight of each indicator in the system, where the comprehensive weight is used to characterize the redundancy and coupling between indicators. The input parameters are shown in Table 2.

The correlation weight $W_{i}^{\prime }$ is calculated by Equation (1).

(1) Wi′=norm(r1,1)norm(R,inf)=∑l=1nril∑l=1nrilmaxj=1nmaxj=1n(∑k=1nrjk)

Comprehensive weight: ${\tilde{W}}_{i}=0.5\left[\left(1-W_{i}^{\prime }\right)+W_{i}\right]$ is sorted according to the size of the comprehensive weight and filtered according to a certain threshold, such as 80%. Therefore, non-critical redundant indicators can be removed.

**Figure 4:**
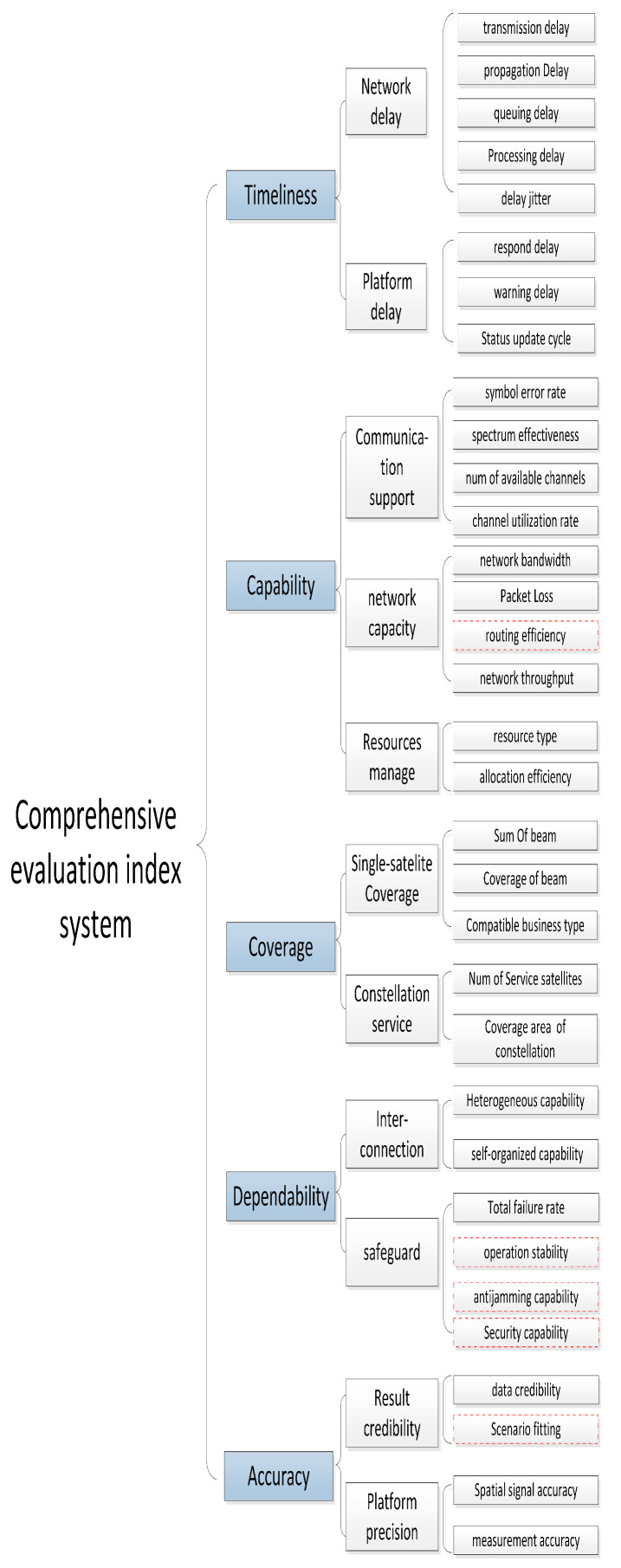
Comprehensive evaluation index system.

Finally, the optimized indicators are mapped to the corresponding elements, and the construction of the comprehensive indicator system is completed. According to the US Department of Defense’s definition of information operations: the indicators with dashed line is measures of effectiveness indicators (MOEI), and the indicators with solid line is measures of performance indicators (MOPI) [30]. The final comprehensive evaluation index system is shown in Figure 4.

## 4. Consistency Enhancement Strategy of Ahp in Group Decision-Making

After completing the construction of the comprehensive index system, determining the weight score of each index in the process of specific evaluation is the next key research content. We score the weight of each index based on the principle of AHP in group decision-making. The scoring matrix obtained by comparing the importance of each indicator pairwise is also called the judgment matrix. The authors of [31] explain how the existing traditional methods would reduce the consistency of the judgment matrix:

1. The expert evaluation scoring input has a certain subjectivity, which could cause the unreliability of the initial input value of the judgment matrix.
2. The discrete integer constraint of the traditional scaling mechanism will further amplify and diffuse the initial input error.

For the first point, even if some experts have strong authority in a certain field, they still inevitably have knowledge limitations. Especially in the context of some interdisciplinary assessments, the subjective limitations of experts become more prominent. In addition, each expert may intentionally make a biased evaluation because of different preferences or representative interests.

Regarding the scaling problem in the second point, we introduce the concept of transitivity for the convenience of explanation [32]. For any indicator a, b, or c, transitivity of importance between indicators can be described by a≻b,b≻c⇒a≻c. Moreover, when a≻b,b≻c, if there are elements in the judgment matrix satisfying c≻a, it indicates that there is a contradictory evaluation in the judgment matrix. In AHP, the detection of transitivity and the ranking of index importance are also a method to find the contradictory elements of the judgment matrix. In addition, this often means that it is difficult for the traditional scale to accurately describe the importance between indicators during the optimization process of the judgment matrix. For example, whena≻b≻d,a≻c≻d,ifa/d=3and b≻c. At this time, it is inappropriate to set both the b/d and c/d scales to 2 due to the [1–9] integer scale—the classic scale in the AHP [33]. It is more suitable to set b to 2.5. Therefore, during the processing of the judgment matrix in this paper, the fractional scale will not be immediately reduced to the integer scale, thereby weakening the influence of the scale on the consistency.

**Figure 5:**
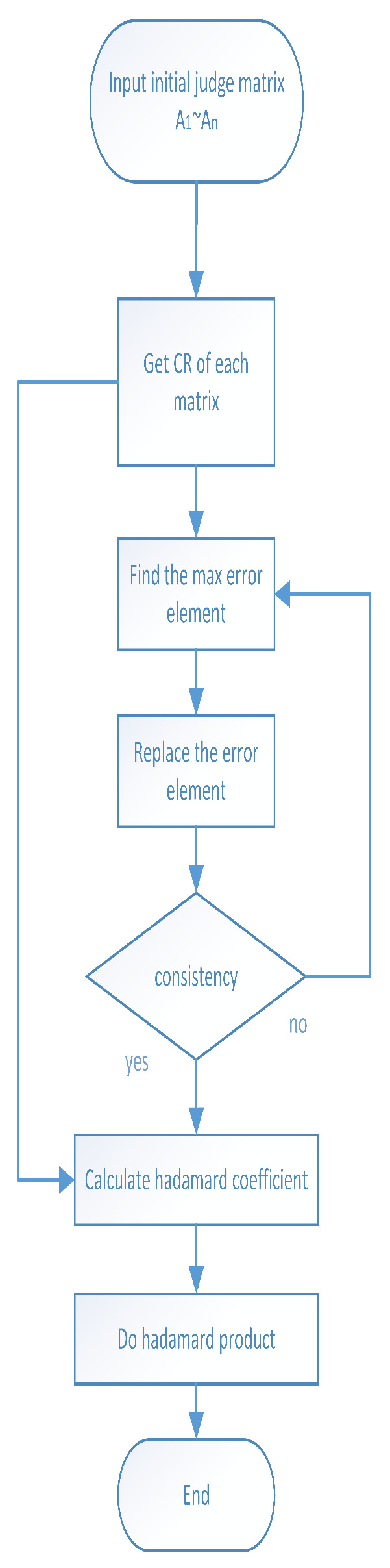
The flow chart of this enhancement strategy.

Based on the above analysis, we decompose the AHP consistency problem in the group evaluation scenario into two sub-problems: (1) How to enhance the consistency of the judgment matrix that does not meet the consistency conditions; (2) how to effectively aggregate judgment matrices from multiple experts. For problem 1, we propose a consistency correction strategy with a better initial value and a faster decline. For problem 2, we give a method for generating the aggregation coefficient combining expert authority and initial deviation. The overall group evaluation strategy process is illustrated in Figure 5.

### 4.1. A Consistency Correction Strategy Based on Improved Eigenvector Method

According to the foregoing description, the key solution of consistency enhancement of the judgment matrix is finding error elements and correcting them based on the existing information. The correction strategy proposed in [31] is only a single correction that could not achieve iterative correction, so the correction effect is limited. Therefore, based on the feature vector method and the theorem of geometric mean induced bias matrix (GMIBM), this paper proposes a consistency correction strategy with better initial value and faster decline. The new strategy relies on the following theoretical foundations.

Perron–Frobenius theorem describes the eigenvalues and eigenvector properties of positive real number matrices. As a special kind of positive real matrix, the positive and negative reciprocal matrix is inherited. As the analytic hierarchy process is an evaluation method constructed based on the positive and negative matrix eigenvectors, the judgment matrix of AHP should also satisfy the PF theorem and related inferences. This theorem is also the theoretical basis for the establishment of the eigenvector method. The PF theorem is described as follows.

**Theorem** **1.** *For a given positive matrix A, the only positive vector x and only positive constant c that satisfy Ax=cx is a vector x that is a positive multiple of the Perron vector (principal eigenvector) of A, and the only such c is the Perron value (principal eigenvalue) of A.*

The authors of [21] give three properties and their demonstrations of a perfectly consistent positive reciprocal matrix. These properties provide a theoretical basis for the correction of the error elements of the judgment matrix, and give a new error matrix description method-GMIBM. Based on that, this paper introduces the valuation of GMIBM as the initial value of the revised strategy.

Based on the above theory, this paper proposes a consistency correction strategy based on eigenvector method with better initial elements. Compared with the previous confidence-based strategy in [31], there are two major advantages: (1) faster descent speed—the speed of the modified matrix’s eigenvalues approaching perfect consistency eigenvalues is faster; (2) stronger adaptability for the judgment matrix with missing elements during rapid assessment. The core idea is to obtain the maximum deviation element through the search of the error matrix. The missing element is automatically determined as the maximum deviation/error element. Then the maximum error element is given a better initial value based on the geometric mean. Finally, the feature vector method iteration loop is enabled until the consistency ratio is met. The steps are shown in the Algorithm 1.
**Algorithm 1:** The algorithm pseudocode of improved method.1: 
$\mathrm{Initialize}:\;input\;pairwise\;comparison\;matrix,\;\;\;\;\;A_{0}={\left\{a_{ij}\right\}}_{1\leq i,j\leq n}\;\;if\;\exists a_{ij}=0,\;then\;a_{ij}=\;1\;$
2: 
$\;Calculate\;the\;bias\;matrix,\;\;\;\;\;\;B_{0}={\left\{\sqrt[{n}]{\prod_{k=1}^{n}a_{ik}a_{kj}}\cdot a_{ji}-1\right\}}_{1\leq i,j\leq n}$
3: 
$\;Find\;the\;location\;of\;\max|b_{ij}|\;,\;\;\;\;\;\;\;\;\;\;then\;a_{ij}=\sqrt[{n}]{\prod_{k=1}^{n}a_{ik}a_{kj}}\;,it⇐0$
4: 
$\;Calculate\;the\;consistency\;index\;of\;A_{0},\;\;\;\;\;\;\;\;\;\;\;CI_{0}=\frac{\lambda {}_{\max}-n}{n-1}$
5: 
$\;\mathrm{While}\;CI_{0}>0.1*RI\left(n\right)\;\;\;$

$\;\;\;\;\;\;\;\;Calculate\;the\;\mathrm{principal}\;\mathrm{eigenvector}\;\mathrm{of}A_{0}\;\;\mathrm{Do}\;\mathrm{operation}\;2\;\;$

$\;\;\;\;\;\;\;\;\;Find\;the\;location\;of\;\max|b_{ij}|\;,\;then\;a_{ij}=\frac{w_{i}}{w_{j}}\;\;\mathrm{Do}\;\mathrm{operation}\;4$

endwhile
6: 
$\;Output\;modified\;matrix\;A_{0}$


### 4.2. Optimization of Aggregation Coefficient Based on Hadamard Convex Combination

Because SIN is a complex network that spans multiple fields, some evaluation objects, such as integration test results or technical verification schemes, often require scoring by multiple experts in different professional fields to achieve a scientific and comprehensive evaluation. Moreover, this process can be attributed to the category of group decision-making. In this paper, the aggregation sequence of judgment matrix with AIJ is applied for group AHP. That is to say, the judgment matrix is aggregated first, and then the eigenvector of the comprehensive evaluation matrix is obtained.

Common judgment matrix aggregation methods include additive aggregation based on arithmetic mean and multiplicative aggregation based on geometric mean. This paper chooses the latter as the aggregation method. Because, there is a serious flaw in the additive aggregation method: When the distance between elements of two near-coherent matrices at the same position is large, additive aggregation may increase the error of the matrix eigenvalue after aggregation, and the consistency of the aggregated matrix could not be guaranteed. Theorem 2 shows that multiplicative aggregation based on hadamard convex combination can neutralize eigenvalues better and inherit the consistency of each judgment matrix. The proving process can be found in [24].

**Theorem** **2.** *If judgment matrices $A_{1},A_{2},...,A_{n}$ are consistent, their hadamard convex combination are consistent.*

Hadamard convex combination defined as: $\tilde{A}=A_{1}^{\lambda_{1}}\cdot A_{2}^{\lambda_{2}}\cdot ...\cdot A_{n}^{\lambda_{n}},\sum_{i=1}^{n}\lambda_{i}=1,0<\lambda_{i}<1.$ The scoring of the experts in the group decision-making process is usually independent. On the one hand, considering the existence of authoritative experts, their scores will have a higher weight. On the other hand, it is necessary to correlate the degree of deviation of the consistency of the initial matrix with the weight of the expert, considering that there is a correlation between the cognitive degree and the consistency when the expert gives the judgment matrix. Based on the above reasons, this paper proposes a new weighting coefficient for the hadamard convex combination (also called the hadamard product), which could be described with Equation (2).

(2) λi=αλi1+βλi2,α+β=1,α,β∈R+λi1=1+i*d∑i=1n(1+i*d),λi2=∑i=1nCIiCIi∑i=1nCIiCIi∑i=1n(∑i=1nCIiCIi)∑i=1n(∑i=1nCIiCIi)

*n* is the number of experts, and $\lambda_{i}^{1}$ is the authority coefficient of the i-th expert, which is usually subjectively scored by the evaluator. This paper presents a generation method based on authoritative ranking, which is equivalent to increasing the authoritative coefficient by a given step size d (we could set d=1/n). $\lambda_{i}^{2}$ is the deviation coefficient of the i-th judgment matrix. The smaller the corresponding consistency index CI, the larger the deviation coefficient. At the same time, the distribution coefficients of α and β also provide strong flexibility. In particular, when α = 1 and β = 0, the hadamard coefficient is a single expert authority coefficient, while when α = 0 and β = 1, the hadamard coefficient is determined in full compliance with the initial consistency of each expert. Such an improvement factor not only reflects the authority of experts, but also takes into account the consistency of the comprehensive matrix.

## 5. Comprehensive Evaluation Example and Numerical Simulation

To prove the rationality and usability of proposed consistency enhancement strategy, the method proposed in this paper is applied to the key part of this evaluation example—evaluation of two generation TDRSS. There are two reasons to choose the TDRSS as the evaluation object: (1) the demonstration platform based on TDRSS is still in the deployment stage, and it faces the upgrading of the system; (2) the TDRSS’s efficiency greatly affects the performance upper limit of the space information network. Therefore, it is obviously a more reasonable choice to evaluate an actual on-orbit system. The main technical elements of the next-generation TDRSS upgrade include laser links, flexible modulation, and delay-tolerant network protocols. Once implemented, the TDRSS’s transmission performance and network capacity will be greatly improved.The specific procedure of this evaluation example is as follows.

**Figure 6:**
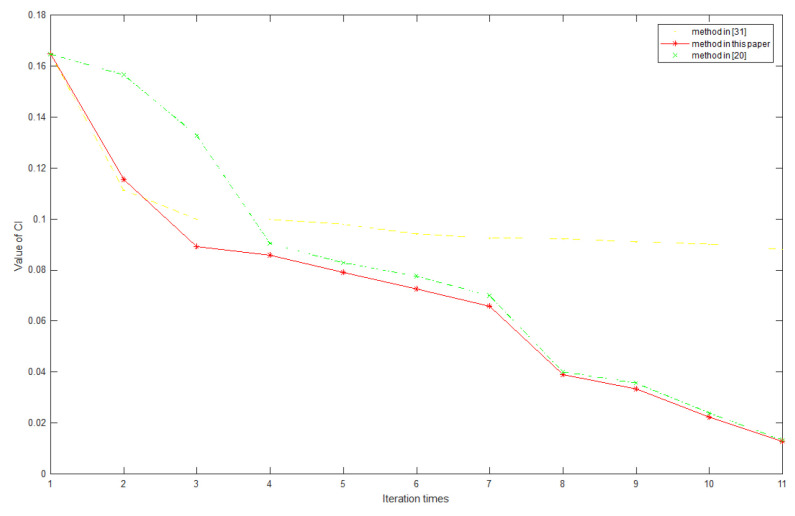
Comparison diagram of three consistency improvement methods.

**Figure 7:**
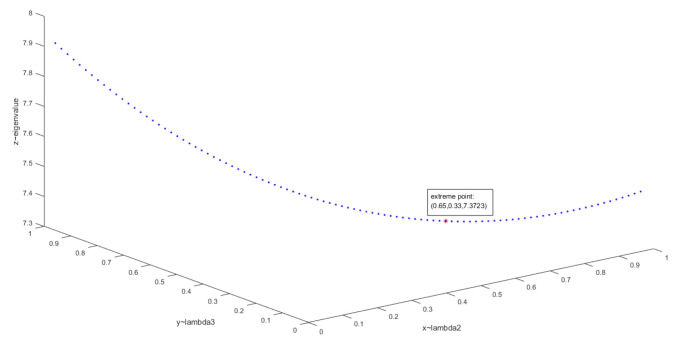
Eigenvalue trend of two matrices’s convex combination.

**Figure 8:**
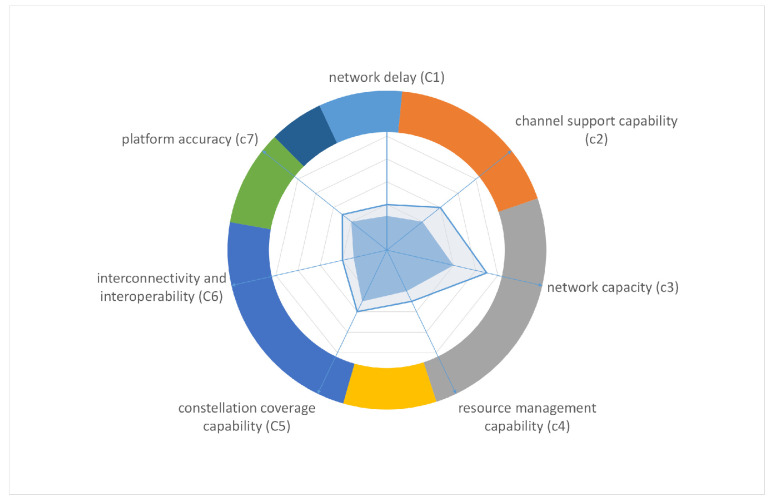
The effectiveness comparison in radar chart.

1. Establish the evaluation goals and build an evaluation model. To simplify the example, this paper only selects a single-level indicator element model.
2. Select relevant element indicators from the comprehensive indicator system. First, enumerate the element-level indicators related to the relay satellite system in the comprehensive indicator system, including network delay (c1), channel support capability (c2), network capacity (c3), resource management and control capability (c4), constellation coverage capability (c5), interconnectivity and interoperability (c6), and platform accuracy (c7).
3. Input the expert group’s score for each indicator to construct a judgment matrix. We set the number of experts to four, and the corresponding expert judgment matrix is $A_{k}$, k = 1 to 4, where each judgment matrix element aijk=cicicjcj1≤i,j≤7
$\begin{array}{c} A_{1}=\left[\begin{array}{ccccccc} 1 & 1/2 & 1/8 & 1/2 & 1/3 & 1 & 2\\ 2 & 1 & 1/5 & 2 & 1/3 & 2 & 1\\ 8 & 5 & 1 & 5 & 4 & 3 & 7\\ 2 & 1/2 & 1/5 & 1 & 2 & 1 & 3\\ 3 & 3 & 1/4 & 1/2 & 1 & 3 & 2\\ 1 & 1/2 & 1/3 & 1 & 1/3 & 1 & 2\\ 1/2 & 1 & 1/7 & 1/3 & 1/2 & 1/2 & 1 \end{array}\right],A_{2}=\left[\begin{array}{ccccccc} 1 & 1/3 & 1/5 & 2 & 1/7 & 1/2 & 1\\ 3 & 1 & 1/2 & 4 & 1/2 & 3 & 2\\ 5 & 2 & 1 & 3 & 2 & 3 & 4\\ 1/2 & 1/4 & 1/3 & 1 & 1/2 & 1/3 & 1\\ 7 & 2 & 1/2 & 2 & 1 & 4 & 5\\ 2 & 1/3 & 1/3 & 3 & 1/4 & 1 & 3\\ 1 & 1/2 & 1/4 & 1 & 1/5 & 1/3 & 1 \end{array}\right],\\ A_{3}=\left[\begin{array}{ccccccc} 1 & 2 & 1/2 & 4 & 1/3 & 1 & 5\\ 1/2 & 1 & 2 & 2 & 3 & 4 & 3\\ 2 & 1/2 & 1 & 1 & 1/3 & 2 & 3\\ 1/4 & 1/2 & 1 & 1 & 1/2 & 2 & 2\\ 3 & 1/3 & 3 & 2 & 1 & 3 & 6\\ 1 & 1/4 & 1/2 & 1/2 & 1/3 & 1 & 2\\ 1/5 & 1/3 & 1/3 & 1/2 & 1/6 & 1/2 & 1 \end{array}\right],A_{4}=\left[\begin{array}{ccccccc} 1 & 1/2 & 1 & 1/3 & 1/5 & 1/2 & 1\\ 2 & 1 & 1 & 4 & 1/2 & 2 & 4\\ 1 & 1 & 1 & 2 & 2 & 3 & 5\\ 3 & 1/4 & 1/2 & 1 & 1/3 & 1 & 2\\ 5 & 2 & 1/2 & 3 & 1 & 2 & 5\\ 2 & 1/2 & 1/3 & 1 & 1/2 & 1 & 3\\ 1 & 1/4 & 1/5 & 1/2 & 1/5 & 1/3 & 1 \end{array}\right] \end{array}$
4. In this step, the maximum real eigenvalue and consistency ratio will be calculated, and the numerical validation of consistency improvement strategy in Section 4.1 will be performed. If there is a matrix with a consistency ratio > 0.1, the consistency will be enhanced by the enhancement strategy proposed in Section 4.1. Taking the A3 matrix as an example, the method proposed in this paper is compared with the method in [20,31]. It can be seen that the method in this paper has a better initial value than the classic eigenvector method [20], which has a faster decline rate than the correction method based on confidence [31], and is not limited by the condition of the matrix element is incomplete. The comparison diagram is shown in Figure 6.
  Analysis of results: The consistency enhancement method previously proposed in [31] is essentially a one-step local algorithm. Its solution is to derive other elements of the judgment matrix by extracting local key information. However, after a certain maximum deviation element is corrected, the algorithm will be unable to continue to optimize due to lack of global information; as shown in the figure, the subsequent iteration process convergence rate is very slow. The method proposed in this paper is a dynamic global algorithm. The updated element output by the algorithm each time is the global information from the judgment matrix. As long as the current error element is updated and corrected, it means that the global information of the judgment matrix has changed. Therefore, the algorithm will not terminate until it reaches the convergence condition. At the same time, due to the a better initial value, the algorithm in this paper shows faster convergence speed than the classical eigenvector method.
5. Use the aggregation method of Section 4.2 to obtain a comprehensive judgment matrix, and finally obtain the weight of each index to form the next generation relay satellite system development proposal. Parameter setting before aggregation: expert authority coefficient A1>A2>A3>A4, and α = β = 0.5; other parameters are shown in Table 3.
  The comprehensive judgment $\bar{A}$ matrix would be
  $$\begin{array}{c} \bar{A}=A_{1}^{0.2778}\circ A_{2}^{0.2952}\circ A_{3}^{0.1992}\circ A_{4}^{0.2278}\\ =\left[\begin{array}{ccccccc} 1 & 0.5847 & 0.3040 & 1.0387 & 0.2311 & 0.6959 & 1.6706\\ 1.7103 & 1 & 0.5983 & 2.8739 & 0.6384 & 2.5881 & 2.0944\\ 3.2899 & 1.6714 & 1 & 2.5328 & 1.6969 & 2.7672 & 4.6426\\ 0.9628 & 0.3480 & 0.3948 & 1 & 0.6701 & 0.8301 & 1.8242\\ 4.3280 & 1.5665 & 0.5893 & 1.4924 & 1 & 2.9778 & 4.0197\\ 1.4369 & 0.3864 & 0.3614 & 1.2047 & 0.3358 & 1 & 2.4724\\ 0.5986 & 0.4775 & 0.2154 & 0.5482 & 0.2488 & 0.4045 & 1 \end{array}\right] \end{array}$$
  After calculation, the maximum real eigenvalue $\lambda_{max}$ = 7.2075. Then, $\lambda_{max}$ is substituted into the CR calculation formula: $CR=\frac{CI}{RI}=\frac{\lambda_{\max}-n}{n-1}/RI\left(n\right)$; random consistency index (RI) [34] is shown in Table 4.
  The calculated consistency ratio $CR_{1}$ = 0.0258 < 0.1. Therefore, the Comprehensive judgment matrix meets the consistency conditions. The eigenvector of $\lambda_{max}$ is the final index weight, which is W = [0.1846 **0.4041 0.6359** 0.2166 **0.5333** 0.2260 0.1262 ${]}^{T}$. It can be seen that the network capacity(C3), constellation coverage capability(C5), and channel support capability(C2) are the three indicators that the group of experts are most concerned in this evaluation example.
  In addition, for comparison, we calculated the case where only expert weights are considered as aggregation coefficients. That is, $\bar{A}=A_{1}^{0.3077}\circ A_{2}^{0.2692}\circ A_{3}^{0.2308}\circ A_{4}^{0.1923}$, $\lambda_{max}=7.2105,CR_{2}<CR_{1}<0.1$. It can be seen from the comparison that the comprehensive judgment matrix generated according to the aggregation coefficient proposed in Section 4.2 has achieved a better consistency ratio in this example. However, it should be pointed out here that the comprehensive judgment matrix obtained according to the AIJ aggregation order is a typical convex combination. Therefore, after aggregation, the maximum real eigenvalues of the comprehensive judgment matrix $\lambda_{\bar{A}}$ must satisfy $\lambda_{\bar{A}}\leq \min\left(\lambda_{A1},\lambda_{A2},\lambda_{A3},\lambda_{A4}\right)$. Therefore, the method proposed in this paper can make the comprehensive judgment matrix’s consistency stronger when the initial input matrix consistency is poor. When the consistency of the initial input matrix is good, this improvement will become less obvious (as in this example, both aggregation coefficients are acceptable and the difference is very small). In order to explain this conclusion more intuitively, the aggregation coefficients of A1 and A4 are set to 0.1, and the aggregation result is mapped from the 5-dimensional space to the 3-dimensional space. Figure 7 shows the eigenvalue’s trend of A2 and A3’s convex combination.
  The function value in this figure is $f\left(x,y\right)=\max|eig\left(A_{2}^{x}\circ A_{3}^{y}\right)|$; *eig* represents a function to obtain the matrix eigenvalues. Obviously, there must be an optimal aggregation coefficient for the convex combination of multiple judgment matrices. The range of aggregation coefficients proposed in this paper: {x,y|x∈[0.33,0.83],y∈[0.17,0.67]}⊂[0,1]×[0,1]. From this, the aggregation coefficient according to the authority of the experts has a certain degree of uncertainty, which leads to a large fluctuation in the final consistency ratio(CR). Therefore, the aggregation coefficient with initial consistency factor proposed in this paper can decrease the floating of CR, thus increase the probability of obtaining a better solution.
6. Calculate the effectiveness improvement ratio = ($s_{2}\cdot w/s_{1}\cdot w)-1$ = 0.4779. After calculation, the comprehensive effectiveness of the second-generation TDRS system is ~48% higher than that of the first-generation system under given conditions. The effectiveness comparison between iand iisystem is shown in Figure 8 and Table 5.
  Example Summary: This evaluation example is a typical multi-objective comprehensive evaluation process, which shows the complete application process of the group AHP consistency enhancement strategy proposed in this paper. Moreover, through numerical examples, it is proved that the strategy converges faster and has stronger consistency. At the same time, it also provides a model for comprehensive evaluation index system to evaluate the subsystem. In addition, the evaluation results also give the main improvement direction of TDRSS which is the backbone system of the space information network, and give an intuitive description of the performance gap between the two generation TDRSS.

## 6. Conclusions

In the process of evolution from sensorweb to space information network, the core technologies involved in remote sensing, navigation, communication, and other network systems urgently need to be demonstrated and verified by demonstration platform. To satisfy the evaluation needs of the SIN demonstration platform based on TDRSS, this paper proposes a comprehensive evaluation design for this demonstration platform. As the first partial work involving a comprehensive evaluation design, a comprehensive index system is built. A universal method for constructing the comprehensive index system from the initial indicator set to index system is provided, which has certain promotion value. As another important part work of comprehensive evaluation design, we propose an analytic hierarchy process consistency enhancement strategy suitable for group evaluation scenarios. Numerical experiments show that the strategy in this paper has a better initial value than the classical eigenvector method, and thus obtains a faster convergence rate. Through the evaluation example of two generation TDRSS, we verify the feasibility of this evaluation strategy.

The index system construction method and AHP improvement strategy proposed in this paper have certain promotion value and practical significance, and can be used for the comprehensive evaluation of other network systems (such as wireless sensor networks). The next work is to explore the construction of the SIN index system based on this paper’s work. We need to strengthen theoretical research about evaluation method, and try to remove dependence on feature vectors.

## Figures and Tables

**Table 1:** Indicator investigation of various related fields.

	Type	Representative Indicator	Remark
terrestrial network	Classic IP Network	Round-trip Delay, delay jitter, Packet Loss, network connectivity...	IETF:RFC2681 RFC2678 RFC3393 RFC7679 RFC7680
	Mobile communication network	Spectrum efficiency, peak rate, traffic density, mobility ...	ITU, IMT-2020’5g’ promotion group
Space network	Measurement	coverage, Maximum service time ..	
	Remote sensing	imaging quality, revisiting period ...	
	Navigation	Constellation coverage, signal accuracy, service continuity, reliability	international civil aviation organization Standard and Recommended Practices
	communication	Channel utilization, bit error rate, outage probability, coverage	
	Tracking & Data Relay	Plan resource utilization, application conflict rate, response time, coverage	
Other system	Cyber space	Consumption, equipment occupancy, bandwidth, convenience, confidentiality, risk	Metrics for Cyber defenses (NASA)
	Information Operation	Anti-jamming, anti-destructive, compatibility, soft/hard attack ability	

**Table 2:** The input parameters of comprehensive weight.

Indicator		Calculation of Correlation Coefficient					
**Serial Number**	**Weight of Value**	**Measured Value (m Group)**			**Correlation Coefficient Matrix, R**		
A${}_{1}$	W${}_{1}$	n${}_{11}$	...	n${}_{1m}$	r${}_{11}$	...	r${}_{1n}$
A${}_{2}$	W${}_{2}$	n${}_{21}$	...	n${}_{2m}$	r${}_{21}$	...	r${}_{2n}$
...	...	...	...	...	...	...	...
A${}_{n}$	W${}_{n}$	n${}_{n1}$	...	n${}_{nm}$	r${}_{n1}$	...	r${}_{nn}$

**Table 3:** Calculation parameter.

	$A_{1}$	$A_{2}$	$A_{3}$	$A_{4}$
$\lambda_{\max}$	7.6672	7.5153	7.9881	7.6284
CI	0.1112	0.0859	0.1647	0.1047
$\lambda_{i}^{2}$	0.2480	0.3211	0.1675	0.2633
$\lambda_{i}^{1}$	0.3077	0.2692	0.2308	0.1923
$\lambda_{i}$	0.2778	0.2952	0.1992	0.2278

$\lambda_{i}$ is the hadamard aggregation coefficient.

**Table 4:** Random consistency index (RI).

n	3	4	5	6	7	8	9
**RI(n)**	0.52	0.88	1.11	1.25	1.34	1.40	1.48

**Table 5:** The input parameter: score and weight.

	Relative Score		*W*:weight
	$S_{1}$:TDRSi	$S_{2}$:TDRSii	
C1	2	3	0.1846
C2	3	5	0.4041
C3	5	8	0.6359
C4	3	4	0.2166
C5	4	5	0.5333
C6	2	3	0.2260
C7	3	4	0.1262

## References

[1] Butler D. (2006). Everything, everywhere. Nature.

[2] Li D. (2016). On “Internet +” Space-based Information Service. J. Remote Sens..

[3] Li D.R., Shen X., Gong J.Y., Zhang J., Lu J.H. (2015). On construction of china’s space information network. J. Wuhan Univ. (Nat. Sci. Ed.).

[4] Yu Q.Y., Meng W.X., Yang M.C., Zheng L.M., Zhang Z.Z. (2016). Virtual multi-beamforming for distributed satellite clusters in space information networks. IEEE Wirel. Commun..

[5] Qu Z., Zhang G., Hong T., Cao H., Zhang W. (2019). Architecture and Network Model of Time-Space Uninterrupted Space Information Network. IEEE Access.

[6] Sobchak T., Shinners D.W., Shaw H. NASA Space Network Project Operations Management: Past, Present and Future for the Tracking and Data Relay Satellite Constellation. Proceedings of the 2018 SpaceOps Conference.

[7] Fan D., Qiu M., Xu X., Chen M. (2018). Research on the Integration Demonstration System of Space Information Network Based on TDRSS. J. Phys. Conf. Ser..

[8] Su W. (2000). Research on the Theory and Methodology of Multi-Index Comprehensive Evaluation. Ph.D. Thesis.

[9] Canright S., Grabowski B. (1995). Indicator Systems and Evaluation.

[10] Shi R.J., Fan X.C., He Y. (2017). Comprehensive evaluation index system for wind power utilization levels in wind farms in China. Renew. Sustain. Energy Rev..

[11] Jia N., You Y., Lu Y., Guo Y., Yang K. (2019). Research on the Search and Rescue System-of-Systems Capability Evaluation Index System Construction Method Based on Weighted Super network. IEEE Access.

[12] Yu H., Li L., Zhang Z., Jin M. Screening of Effectiveness Evaluation Index and Construction of Network Index System of Command and Control System. Proceedings of the 2019 IEEE 3rd Information Technology, Networking, Electronic and Automation Control Conference (ITNEC).

[13] Elhady A.M. Remote sensing satellite system overall effectiveness analysis and modeling. Proceedings of the Aerospace Conference.

[14] Li H., Li D., Li Y. (2018). A multi-index assessment method for evaluating coverage effectiveness of remote sensing satellite. Chin. J. Aeronaut..

[15] A Series Document of the IP Performance Metrics (IPPM)—6248/7312/7679/7680. https://datatracker.ietf.org/wg/ippm/documents/.

[16] Yang Y., Li X. (2003). Research on IP Network Performance Index System. J. Commun. Syst..

[17] Xie T., Xie D., Guo C., Gao M. The Study on Effectiveness Evaluation Indicator System of Wireless Access System. Proceedings of the International Conference on Robots and Intelligent System-IEEE Computer Society.

[18] Zhang Z., Bian D.-M. (2010). Study on Performance Evaluation Method of Satellite Communication Network. Digit. Commun. World.

[19] Harker P.T. (1987). Derivatives of the Perron root of a positive reciprocal matrix: With application to the analytic hierarchy process. Appl. Math. Comput..

[20] Saaty T.L. (2003). Decision-making with the AHP: Why is the principal eigenvector necessary. Eur. J. Oper. Res..

[21] Ergu D., Kou G., Peng Y., Zhang M. (2016). Estimating the missing values for the incomplete decision matrix and consistency optimization in emergency management. Appl. Math. Model..

[22] Aguarón J., Escobar M.T., Moreno-Jiménez J.M. (2020). Reducing Inconsistency measured by the Geometric Consistency Index in the Analytic Hierarchy Process. Eur. J. Oper. Res..

[23] Lin C., Kou G., Peng Y., Alsaadi F.E. (2020). Aggregation of the nearest consistency matrices with the acceptable consensus in AHP-GDM. Eur. J. Oper. Res..

[24] Zhang Z. (2014). Research on Evaluation Method for Credibility of Simulation System. Ph.D. Thesis.

[25] Moreno-Jiménez J.M., Aguarón J., Escobar M.T. (2008). The Core of Consistency in AHP-Group Decision Making. Group Decis. Negot..

[26] Aguarón J., Escobar M.T., Moreno-Jiménez J.M. (2016). The precise consistency consensus matrix in a local AHP-group decision making context. Ann. Oper. Res..

[27] Aguarón J., Escobar M.T., Moreno-Jiménez J.M., Turón A. (2019). AHP-Group Decision Making Based on Consistency. Mathematics.

[28] Janković A., Popović M. (2019). Methods for assigning weights to decision makers in group AHP decision-making. Decis. Mak. Appl. Manag. Eng..

[29] Wu Z., Jin B., Fujita H., Xu J. (2020). Consensus analysis for AHP multiplicative preference relations based on consistency control: A heuristic approach. Knowl.-Based Syst..

[30] Joint Chiefs of Staff (2006). Joint Publication 3-13: Information Operations.

[31] Shi D., Liu F., Fei L., Nie K. Comprehensive Evaluation of Space Information Network Simulation System Based on GEO Satellite. Proceedings of the 2018 14th International Wireless Communications and Mobile Computing Conference (IWCMC).

[32] Gass S.I., Standard S.M. (2002). Characteristics of positive reciprocal matrices in the analytic hierarchy process. J. Oper. Res. Soc..

[33] Saaty T.L., Tran L.T. (2007). On the invalidity of fuzzifying numerical judgments in the Analytic Hierarchy Process. Math. Comput. Model..

[34] Peláez J.I., Martínez E.A., Vargas L.G. (2018). Consistency in Positive Reciprocal Matrices: An Improvement in Measurement Methods. IEEE Access.

